# Adult-onset *PLEC*-related congenital myasthenic syndrome-myopathy overlap with upper limb predominant weakness

**DOI:** 10.1007/s10048-026-00900-8

**Published:** 2026-04-18

**Authors:** Angel Jose, Aina Jasrul Azily, Katie Doyle, Karen Stals, Hayley Lees, Caoimhe McKenna, Amy Jayne McKnight, John McConville, Grace McMacken

**Affiliations:** 1 School of Medicine, Dentistry and Biomedical Sciences, Queen’s University Belfast, Belfast, UK; 2 Department of Neurology, Ulster Hospital, South Eastern Health and Social Care Trust, Belfast, UK; 3 Exeter Genomics Laboratory, Royal Devon University Healthcare NHS Foundation Trust, Exeter, UK; 4 Northern Ireland Regional Genetics Service, Belfast City Hospital, Belfast Health and Social Care Trust, Belfast, UK; 5 Centre for Public Health, Queen’s University Belfast, Belfast, UK; 6 Wellcome-Wolfson Institute for Experimental Medicine, Queen’s University Belfast, Belfast, UK

**Keywords:** *PLEC*, Congenital myasthenic syndrome, Intronic variant, RNA analysis, Genetic diagnosis

## Abstract

Congenital myasthenic syndromes (CMS) are genetically heterogeneous disorders in which precise molecular diagnosis is essential for management. Pathogenic variants in *PLEC* are a rare cause of autosomal recessive CMS and are frequently associated with epidermolysis bullosa simplex (EBS). We report an adult-onset case with EBS from birth, upper limb–predominant weakness, combined myopathic and myasthenic features, and subtle cardiomyopathy. Initial CMS gene panel testing identified a heterozygous canonical splice-site variant in *PLEC*. Subsequent trio exome sequencing revealed a second heterozygous intronic variant in trans, and RNA analysis demonstrated intron retention resulting in a frameshift, enabling variant reclassification and definitive diagnosis. This case expands the phenotypic spectrum of *PLEC*-related disease and highlights the value of transcript-level analysis in resolving cryptic Mendelian disorders.

**Supplementary Information:**

The online version contains supplementary material available at 10.1007/s10048-026-00900-8.

## Introduction

Congenital myasthenic syndromes (CMS) are a heterogeneous group of inherited disorders of neuromuscular transmission caused by pathogenic variants in more than 40 genes [1]. Establishing a molecular diagnosis is critical, as prognosis and treatment response vary significantly by genetic subtype. Pathogenic variants in *PLEC* represent a rare cause of CMS and are inherited in an autosomal recessive manner. In addition to CMS, biallelic *PLEC* variants cause a spectrum of disorders including epidermolysis bullosa simplex (EBS) and myopathy. These phenotypes reflect the essential roles of plectin in maintaining cytoskeletal integrity in mechanically stressed tissues and in post-synaptic neuromuscular junction (NMJ) organisation [2, 3]. Reported cases of *PLEC*-related CMS typically present in infancy or early childhood with dermatological manifestations followed by neuromuscular involvement (Supplementary Table 1) [4–6]. 

Here, we report an unusual adult-onset case of *PLEC*-related CMS with upper limb–predominant weakness, cardiomyopathy and non-diagnostic initial genetic testing. A definitive diagnosis was achieved through RNA analysis of an intronic variant, underscoring the importance of phenotype-driven investigation and functional studies in resolving cryptic Mendelian disease.

## Case presentation

The patient first presented at the age of 26 with a 3-year history of progressive upper limb weakness, predominantly affecting shoulder abduction, elbow flexion and finger and wrist extension, with no ocular, bulbar or respiratory symptoms. The patient had a diagnosis of EBS from birth, but normal developmental and motor milestones. There was no family history of neuromuscular problems and no consanguinity.

**Fig. 1:**
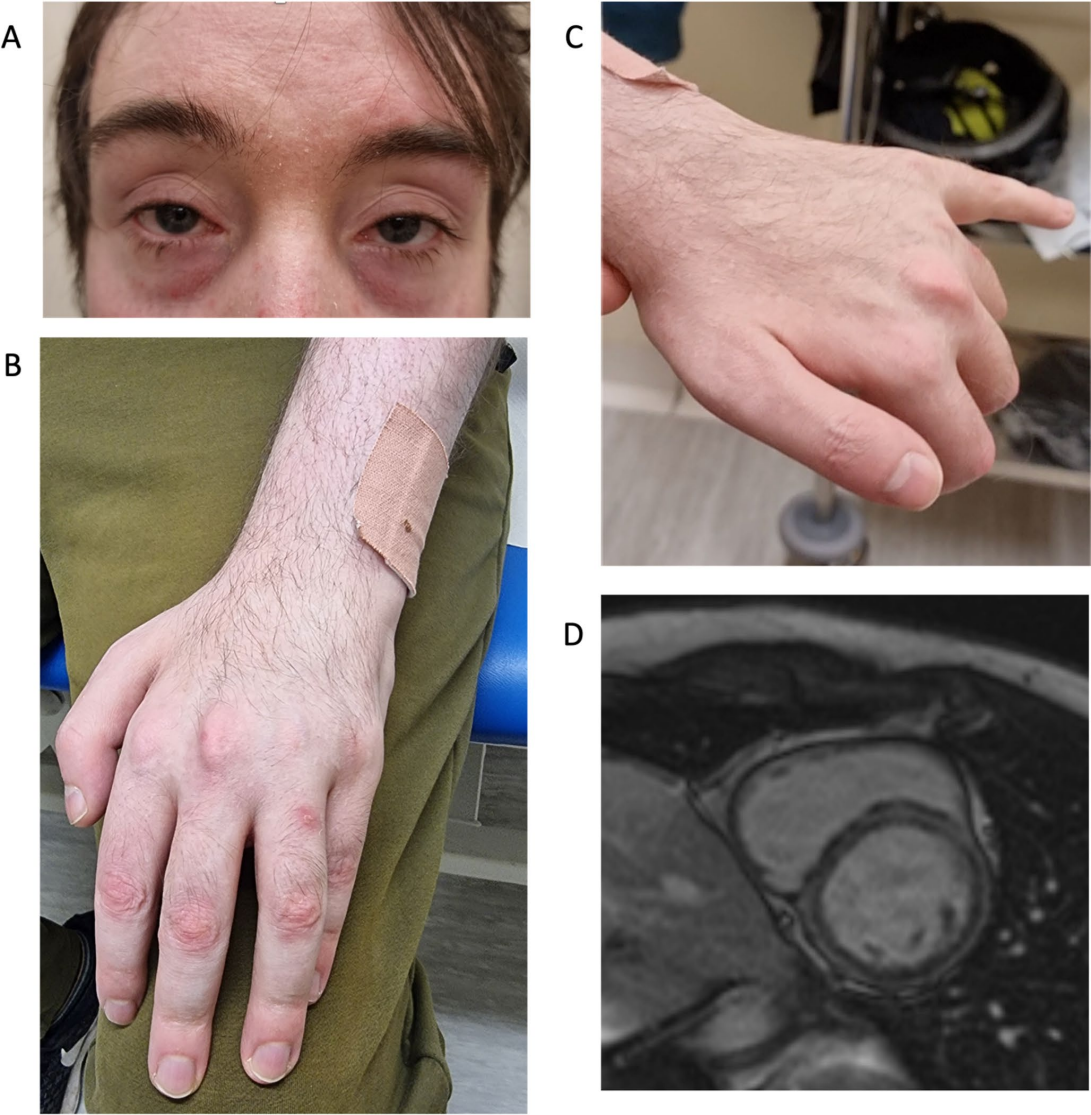
Clinical features of *PLEC* CMS. (**a**) Bilateral ptosis (fatiguable); (**b**, **c**) Upper limb weakness, wasting of intrinsic hand muscles, and finger and wrist drop (also fatiguable) mimicking slow channel CMS; (**d**) Late gadolinium enhancement cardiac magnetic resonance imaging demonstrating a non-ischaemic pattern of myocardial fibrosis with ring-like enhancement at basal and mid-ventricular levels. Informed consent was obtained for the publication of images

On examination there was mild fatiguable ptosis, bilateral symmetrical weakness of eye and mouth closure (Fig. 1). Symmetric proximal-predominant upper limb weakness was present (shoulder abduction MRC (medical research council) grade 3/5, elbow flexion 2/5, elbow extension 4/5, finger extension ranging from 1/5 at the index finger to 4/5 at 5th finger), with reduced upper limb deep tendon reflexes. Lower limb strength was preserved (MRC 5/5 throughout). Sensation, axial strength and gait were all normal. Over the following 6 years, he developed mild ophthalmoparesis and progressive facial and upper limb weakness, while lower limb strength remained entirely preserved. Bulbar function and respiratory function (as assessed by forced vital capacity (FVC) and forced expiratory volume in one second (FEV1)) remained normal.

Nerve conduction studies showed markedly reduced median and ulnar compound muscular action potential (CMAP) amplitudes and moderately reduced peroneal responses with preserved velocities and normal sensory studies. Electromyography (EMG) demonstrated spontaneous activity and small amplitude, long duration polyphasic motor units with early recruitment in bilateral biceps brachii, right vastus lateralis and right tibialis anterior muscles. Repetitive nerve stimulation (RNS) was carried out at 3 Hz in abductor pollicis brevis only, due to poor patient tolerance. This revealed a borderline 10–11% decrement, suggestive of an NMJ defect. There was no post-exercise facilitation, and no double CMAPs were observed. Creatinine kinase (CK) was elevated at 1757 U/L. Magnetic resonance imaging (MRI) of the brain and spine were normal, muscle MRI was not performed. Cardiac investigations revealed multiple ventricular ectopic beats on ECG. Echocardiography demonstrated a dilated left ventricle with mildly reduced systolic function (LVEF 56%). Cardiac MRI demonstrated late gadolinium enhancement consistent with non-ischaemic myocardial fibrosis (Fig. 1d).

**Fig. 2:**
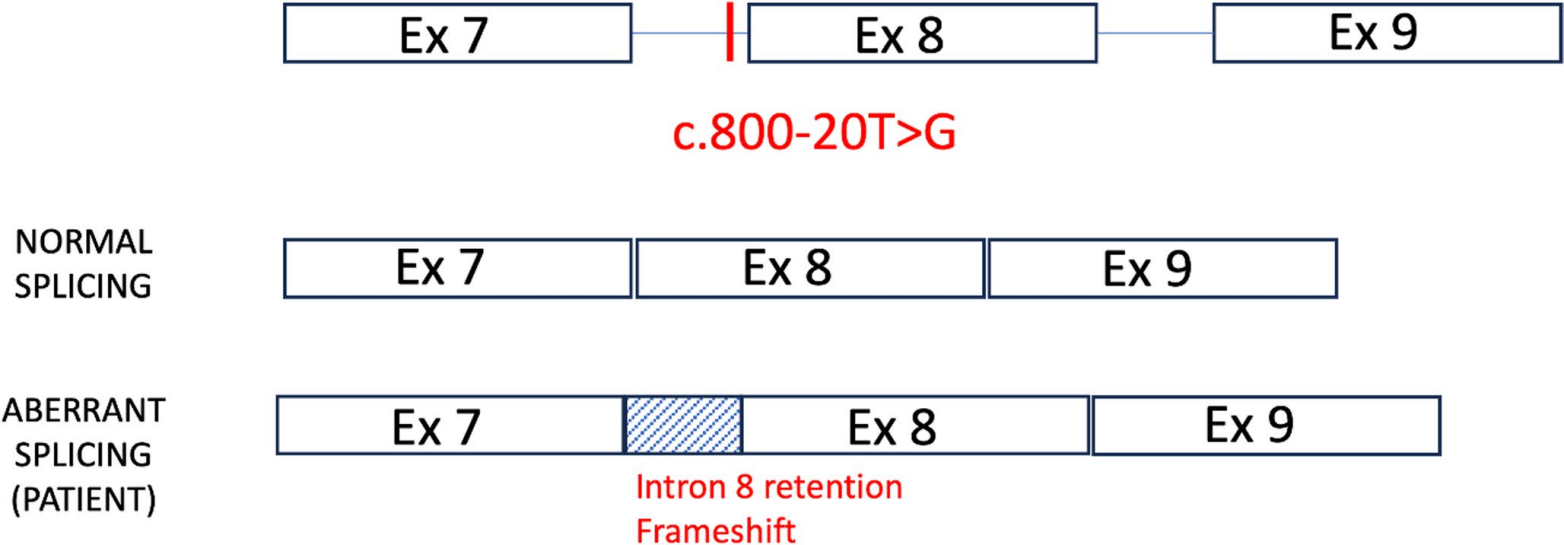
Intronic *PLEC* variant causes aberrant splicing. Schematic representation of *PLEC* exons 7–9 showing the location of the c.800-20T > G variant within intron 8. Normal splicing generates an in-frame transcript, whereas the c.800-20T > G variant results in intron 8 retention, causing a frameshift and predicted premature termination

Given the combination of myopathy, NMJ dysfunction and epidermolysis bullosa simplex, a plectinopathy was considered. Targeted CMS gene panel testing (R80 CMS panel including 27 genes, listed in Supplementary Table 2) was performed and identified a single heterozygous canonical splice-site variant in *PLEC* (NM_000445.5 c.3162 + 1G > A), initially reported as a variant of uncertain significance (VUS) in the context of a single heterozygous finding without confirmed biallelic disease. Chromosomal microarray analysis was normal. Subsequent trio exome sequencing and gene-agnostic trio analysis confirmed paternal inheritance of this variant and identified a second, maternally-inherited intronic *PLEC* variant (NM_000445.5 c.800-20T > G). Although located 20 bp upstream of exon 8, this variant was not reported on the initial targeted CMS panel, reflecting limitations in intronic coverage and analysis of non-canonical splice-region variants in panel-based testing. Despite its rarity in population databases, at the time of analysis this variant was initially classified as a VUS in the absence of functional evidence (according to ACGS 2020 variant classification guidelines) [7]. Owing to strong clinical suspicion, RNA analysis was undertaken and demonstrated intron 8 retention resulting in a frameshift, p.(Asp267Glyfs*60) (Fig. 2). Variant interpretation was performed according to ACMG/AMP guidelines (Richards et al., 2015) and ACGS 2024 recommendations [8, 9]. The canonical splice-site variant NM_000445.5:c.3162 + 1G > A affects the + 1 donor position and is consistent with loss-of-function, a known disease mechanism for *PLEC*. It is absent from gnomAD and was identified in trans with a second variant. The intronic variant NM_000445.5:c.800-20T > G was shown by RNA analysis to result in intron 8 retention and a frameshift (p.(Asp267Glyfs*60)), providing strong functional evidence of pathogenicity. This variant is present at very low frequency in gnomAD (3 heterozygotes, no homozygotes) [10]. This supported pathogenicity of the intronic variant and of biallelic disease-causing variants in *PLEC*.

Treatment with pyridostigmine was ineffective. Salbutamol treatment resulted in gradual clinical improvement over 6 months, characterised by subjective improvement in upper limb endurance and improvement in MRC grades in proximal upper limb muscles.

## Discussion

*PLEC*-related CMS is an ultra-rare postsynaptic CMS subtype that usually presents in infancy or early childhood, often in association with epidermolysis bullosa simplex (EBS). The present case expands the recognised clinical spectrum by demonstrating adult-onset disease with a striking upper limb–predominant pattern of weakness, slow progression, and persistent sparing of the lower limbs despite prolonged follow-up. Although CMS are phenotypically heterogeneous, this distribution of weakness is uncommon within the recognised spectrum. One recent report described adult-onset myopathy with distal upper limb weakness due to *PLEC* mutations, but without evidence of NMJ dysfunction, making the present case clinically distinct and supporting phenotypic overlap between myopathic and myasthenic manifestations of *PLEC*-related disease [11]. 

Several findings supported a CMS diagnosis in this patient. These included fatiguable weakness, a reproducible decrement on repetitive nerve stimulation, and clinical improvement following salbutamol. Prior ultrastructural and electrophysiological studies in *PLEC*-related CMS have demonstrated reduced miniature endplate potentials and progressive destruction of postsynaptic junctional folds, consistent with a postsynaptic defect [12]. The response to salbutamol in this case is consistent with reported therapeutic responses in other postsynaptic CMS subtypes and with in vivo studies demonstrating partial restoration of postsynaptic architecture, including junctional folds [13]. At the same time, the coexistence of hyperCKaemia and myopathic EMG changes supports concomitant structural muscle involvement, illustrating that *PLEC*-related disease cannot be considered purely synaptic or purely myopathic.

Plectin is a cytolinker protein essential for maintaining structural integrity in tissues exposed to mechanical stress, including skeletal and cardiac muscle, skin, and the NMJ [14]. Consistent with this multifunctional role, pathogenic variants in *PLEC* give rise to a spectrum of overlapping phenotypes encompassing epidermolysis bullosa simplex (EBS), myopathy, cardiomyopathy, and CMS [2]. In this context, the presence of EBS in this patient provided an important clinical clue prompting consideration of *PLEC*-related disease.

Cardiac findings further broadened the phenotype. Although cardiac involvement is not typical of most CMS subtypes, it has been reported in plectinopathies, particularly in EBS–muscular dystrophy overlap syndromes [4]. In this case, cardiomyopathy was clinically silent and detected only through targeted investigation: echocardiography showed mildly reduced left ventricular systolic function, while cardiac MRI demonstrated late gadolinium enhancement consistent with myocardial fibrosis. These findings support systematic cardiac assessment in individuals with suspected *PLEC*-related disease, including echocardiography and, where indicated, cardiac MRI. This may be particularly relevant when β2-agonists are used symptomatically, given their potential cardiovascular effects.

This case also highlights the diagnostic limitations of standard genetic testing approaches. Initial targeted CMS panel testing identified only a single *PLEC* splice-site variant, whereas trio exome sequencing identified a second non-canonical intronic variant. RNA analysis then demonstrated intron 8 retention with frameshift, supporting pathogenicity and resolving the molecular diagnosis. This illustrates the value of combining phenotype-driven suspicion with broader sequencing strategies and transcript-level analysis when non-canonical variants are suspected. Such approaches are important not only for diagnosis, but also for directing surveillance, treatment decisions, genetic counselling, and readiness for future therapeutic trials.

## Supplementary Information

Below is the link to the electronic supplementary material.

Supplementary Material 1 (DOCX 19.2 KB)

## Data Availability

No datasets were generated or analysed during the current study.
