# Supplementary material for: Adult-onset PLEC-related congenital myasthenic syndrome-myopathy overlap with upper limb predominant weakness
Source: Neurogenetics. 2026 Apr 18;27(1):31. doi: 10.1007/s10048-026-00900-8 (PMC13091873; doi:10.1007/s10048-026-00900-8)
Supplement: Supplementary file 1 — Supplementary Material 1 (DOCX 19.2 KB) [file 10048_2026_900_MOESM1_ESM.docx]

| Reference | cDNA variant | Protein change | Exon/Intron | Variant type |
| --- | --- | --- | --- | --- |
| Banwell et al.(16) | c.6169C>T | p.(Gln2057Ter) | Exon 31 | Nonsense |
|  | c.12043dupG | p.(Glu4015GlyfsTer6) | Exon 32 | Frameshift |
| Selcen et al.(13) | c.6955C>T | p.(Arg2319Ter) | Exon 31 | Nonsense |
|  | c.12043dupG | p.(Glu4015GlyfsTer6) | Exon 32 | Frameshift |
| Forrest et al.(5) | c.10187delTGTC | p.Val3396AlafsTer11 | Exon 32 | Frameshift |
|  | IVS11+2T>G | p.? | Intron 11 | Splice donor |
| Gonzales-Garcia et al.(17) | c.3086G>A | p.(Arg1029His) | Exon 25 | Missense |
|  | c.9679_9766del | p.(Asp3229ValfsTer21) | Exon 25 | Frameshift |
| Mroczek et al.(18) | c.1_9del (homozygous) | p.(Met1_Gly3del) | Exon 1 | In-frame deletion |

Supplemental Table 1: Summary of previously published variants in PLEC associated with Congenital Myasthenic Syndrome (CMS), (GenBank reference no: NM_000445).

| *AGRN* | *CHAT* | *GFPT1* | *SCN4A* |
| --- | --- | --- | --- |
| *ALG2* | *CHRNA1* | *GMPPB* | *SLC18A3* |
| *ALG14* | *CHRNB1* | *LRP4* | *SLC25A1* |
| *CHRND* | *CHRNE* | *MUSK* | *SLC5A7* |
| *CHRNG* | *COL13A1* | *MYO9A* | *SYT2* |
| *COLQ* | *DOK7* | *PLEC* | *TOR1AIP1* |
| *DPAGT1* | *RAPSN* | *VAMP1* |  |

Supplementary Table 2: List of 27 genes included in the initial CMS gene panel
